# Effects of Endotracheal Tube with Adhesive Superficial Laryngeal Electrodes for Intraoperative Nerve Monitoring on Laryngopharyngeal Complications during Thyroidectomy

**DOI:** 10.3390/biomedicines11092544

**Published:** 2023-09-15

**Authors:** Jiae Moon, Jin Kyong Kim, Hye Jung Shin, Jooeun Park, Na Young Kim, Kee-Hyun Nam

**Affiliations:** 1 Department of Anesthesiology and Pain Medicine, Anesthesia and Pain Research Institute, Yonsei University College of Medicine, Seoul 03722, Republic of Korea; answldo@yuhs.ac (J.M.); rlo2@yuhs.ac (J.P.); 2 Department of Surgery, Yonsei University College of Medicine, Seoul 03722, Republic of Korea; jkkim3986@yuhs.ac; 3 Biostatistics Collaboration Unit, Department of Research Affairs, Yonsei University College of Medicine, Seoul 03722, Republic of Korea; hjshin105@yuhs.ac

**Keywords:** thyroid cancer, thyroidectomy, intraoperative neural monitoring, postoperative sore throat, hoarseness, cough

## Abstract

The endotracheal tube (ETT) with laryngeal adhesive electrodes for intraoperative neural monitoring (IONM) may be related to laryngopharyngeal complications, such as postoperative sore throat (POST), hoarseness, and coughing. We aimed to evaluate the effects of the ETT with laryngeal adhesive electrodes for IONM on the occurrence of laryngopharyngeal complications during thyroidectomy. In this retrospective study, we included 176 patients who underwent thyroidectomy for thyroid cancer between September 2020 and February 2021. The patients were categorized into control (*n* = 108) and IONM (*n* = 68) groups. Patients in the IONM group were intubated with the ETT with surface electrodes. Characteristics of the patients and surgery, perioperative variables, and laryngopharyngeal complications, including POST, hoarseness, and cough, were evaluated. The severity and incidence of POST were comparable between the two groups on postoperative days 0, 1, and 2 (*p* = 0.103, 0.386, and 0.056, respectively). Furthermore, no significant differences were observed in the occurrence of postoperative hoarseness and cough between the groups. The ETT with laryngeal adhesive electrodes for IONM during thyroidectomy did not affect the incidence and severity of postoperative laryngopharyngeal complications, including POST, hoarseness, and cough. Further prospective, double-blinded, randomized clinical trials are required to gain a clearer understanding.

## 1. Introduction

Intraoperative neural monitoring (IONM) in thyroidectomy reduces the incidence of recurrent laryngeal nerve (RLN) injury by facilitating RLN identification compared to conventional visual nerve identification and has gained widespread acceptance [1]. IONM is achieved by intraoperatively stimulating nerves and evaluating laryngeal muscle movement using electromyography (EMG) [2]. One of the most common methods involves surface electrodes on an endotracheal tube (ETT). Two types of commercially available ETT are the electrode-embedded EMG tube and the laryngeal adhesive electrode that utilizes EMG electrodes attached in a patch-like manner to the ETT using adhesive tape [3].

Postoperative sore throat (POST), hoarseness, and cough are the commonly reported laryngopharyngeal complications associated with endotracheal intubation (ETI) [4]. ETI-related parameters such as tube diameter, cuff pressure, cuff design, and intubation technique are major risk factors for laryngopharyngeal side effects [5,6,7]. Notably, the manipulation of thyroid tissue near the trachea and movement of the ETT within the trachea by neck hyperextension during thyroidectomy increase the risk of laryngopharyngeal complications, especially POST [8,9,10]. Consequently, these complications are more common in thyroidectomy than in other surgeries [5,8,11,12]. These side effects are undesirable and may compromise the quality of recovery by reducing patient satisfaction and prolonging the recovery period [9,13,14].

The ETT with adhesive surface electrodes used for IONM during ETI may pose a higher risk of mechanical irritation due to differences in morphological and/or mechanical characteristics compared to the standard ETT [3]. Although the laryngeal adhesive electrode may be related to laryngopharyngeal complications by influencing the resistance or stiffness of the ETT surface, studies elucidating its effect on postoperative laryngopharyngeal complications are lacking. Thus, we aimed to evaluate the impact of the ETT with laryngeal adhesive electrodes for IONM during thyroidectomy on the occurrence of laryngopharyngeal complications, including POST, hoarseness, and cough.

## 2. Materials and Methods

### 2.1. Patient Population

**Figure 1:**
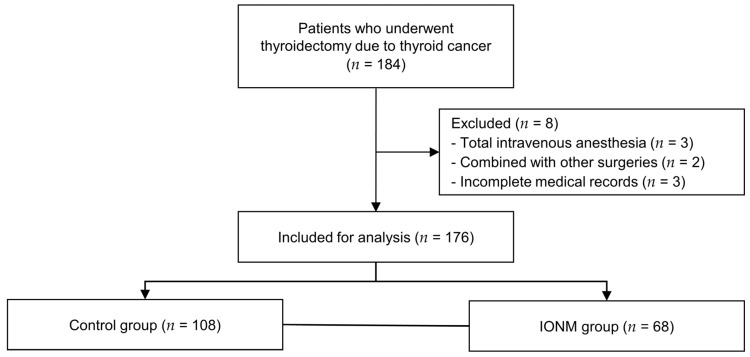
Flow diagram of the study. IONM, intraoperative neural monitoring.

We identified the electronic medical records of 184 patients who underwent elective thyroidectomy for thyroid cancer between September 2020 and February 2021 at Severance Hospital, Seoul, Korea. Thyroid nodules were preoperatively diagnosed based on findings from ultrasonography-guided fine needle aspiration biopsy and neck computed tomography. The decision regarding the necessity of surgery for thyroid nodules and the extent of thyroid resection was determined based on the American Thyroid Association guidelines [15]. All patients included in this study underwent initial thyroid surgery, while those who underwent surgery for recurrent thyroid cancer were excluded. Moreover, patients who received total intravenous anesthesia (*n* = 3), underwent other combined operations (*n* = 2), or had incomplete medical records (*n* = 3) were excluded. Consequently, 176 patients were included in the final analysis and were categorized into the control (*n* = 108) or IONM (*n* = 68) group (Figure 1).

### 2.2. Intraoperative Neural Monitoring

**Figure 2:**
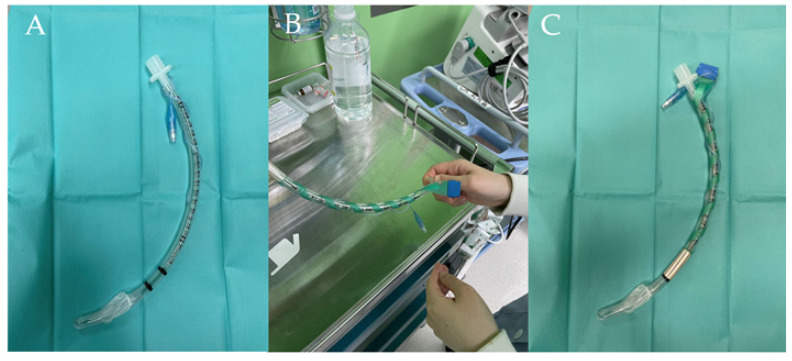
(**A**) An endotracheal tube without laryngeal adhesive electrodes was used for patients in the control group; (**B**) Laryngeal adhesive electrodes were attached to the endotracheal tube; (**C**) An endotracheal tube with laryngeal adhesive electrodes was used for patients in the IONM group. IONM, intraoperative neural monitoring; POST, postoperative sore throat; PACU, post-anesthetic care unit; POD, postoperative day.

The ETT with surface electrodes (Inomed Laryngeal Electrode, Emmendingen, Germany) was placed in contact with the true vocal cords (Figure 2). IONM was performed using a C2 NerveMonitor (Inomed, Germany). After the neck of the patient was extended and the position was adjusted, adequate contact between the electrodes and the vocal cords was confirmed by checking the signals from the C2 NerveMonitor system.

### 2.3. Indications for Intraoperative Neural Monitoring

According to the Health Insurance Review and Assessment Service (HIRA), the indications for IONM include recurrent thyroid cancer in the central region; preoperative unilateral vocal cord paralysis; thyroid cancer with clear lymph node metastasis in the central region; suspected or confirmed extrathyroidal extension (T3b) in thyroid cancer; and high-risk factors for thyroid surgery, such as Graves’ disease or marked thyroid enlargement, as well as undergoing parathyroid surgery. The decision on whether to employ IONM for all patients was made according to HIRA’s indications, with no personal judgment by the physician.

### 2.4. Anesthesia

General anesthesia was administered according to the standard protocol of our institution [16]. Upon the arrival of patients to the operating room, noninvasive blood pressure, electrocardiogram, oxygen saturation, and patient state index (PSI, SedLine^®^, Masimo Corp., Irvine, CA, USA) were monitored. Patients were pre-oxygenated for 3 min using a mask with 100% oxygen and premedicated with 0.1 mg of glycopyrrolate. Anesthesia was induced by administering propofol (1–2 mg/kg) and remifentanil (0.05–0.1 µg/kg), followed by rocuronium (0.6 mg/kg) to facilitate tracheal intubation. After intubation, the endotracheal tube cuff pressure was adjusted to approximately 25 cmH_2_O using a manometer. Patients were mechanically ventilated with a positive end-expiratory pressure of 5 cmH_2_O and a tidal volume of 6–8 mL/kg to maintain the end-expiratory carbon dioxide between 35 and 40 mmHg. Anesthesia was maintained with sevoflurane (0.6–1.0 age-adjusted minimum alveolar concentration) and remifentanil at 0.03–0.1 μg/kg/min, targeting a PSI of 40–60. At the end of the surgery, we intravenously administered sufentanil (1 µg/kg; BCWorld Pharm Co., Ltd., Seoul, Republic of Korea) for postoperative pain management and ramosetron (0.3 mg; Nasea^®^, Astellas Pharma Korea, Seoul, Republic of Korea) for the prevention of nausea and vomiting. In the post-anesthesia care unit (PACU), postoperative pain was measured using a numeric rating scale (NRS) of 0–10 points, and 1 µg/kg of fentanyl was administered in cases where the score was ≥4. Nausea and vomiting were evaluated on a scale of 0–3 (0 = none, 1 = mild, 2 = moderate, and 3 = severe), and metoclopramide (10 mg) was administered in severe cases.

### 2.5. Surgical Procedure

All patients underwent open thyroidectomy, with surgery types including open conventional thyroidectomy [17] or minimally invasive thyroidectomy (MIT) [18]. Patients were placed in a supine position with the neck extended after general anesthesia.

During open conventional thyroidectomy, a transverse collar incision of approximately 5–6 cm in length was made in the midline of the anterior neck, 2 cm above the sternal notch. The subcutaneous tissue and platysma muscle were separated using electrocautery. A subplatysmal skin flap was created to achieve adequate working space from the sternal notch to the hyoid bone level superiorly and across both medial sides of the anterior border of the sternocleidomastoid muscle (SCM) laterally. The strap muscles were divided at the midline, and the areolar tissue was dissected to expose the thyroid.

During MIT, a skin incision of approximately 2–3 cm was made along the skin crease on the main lesion side of the lower neck. Skin flaps were created after the platysma muscle was divided using electrocautery. The medial border of the SCM and the lateral border of the strap muscle were exposed using electrocautery. Moreover, the thyroid gland was exposed under the lateral border of the strap muscles. Both open conventional thyroidectomy and MIT were performed with ligation of the superior and inferior thyroid vessels after the thyroid gland was exposed to preserve the parathyroid glands and RLNs.

The surgical extent included thyroid lobectomy, ipsilateral total and contralateral partial thyroidectomy, and bilateral total thyroidectomy. All bilateral total thyroidectomies were performed using open conventional thyroidectomy. Furthermore, central compartment neck dissection was performed for therapeutic or preventive purposes in all patients.

### 2.6. Data Collection and Outcomes

Data on the characteristics of patients were collected, including sex; age; body mass index (BMI); American Society of Anesthesiologists (ASA) physical status; underlying diseases, such as hypertension, diabetes mellitus, or chronic cough; smoking history; and preoperative symptoms, such as compression or hoarseness. Moreover, data on surgical characteristics were collected, including type of operation; type of thyroidectomy; surgical location; co-operation with parathyroidectomy; intraoperative findings, such as RLN invasion, strap muscle invasion, tracheal invasion, esophageal invasion, thyroiditis, enlarged lymph nodes, and bleeding tendency; and RLN injury. Perioperative data, including the duration of anesthesia and operation; total fluid intake; blood loss; dose of remifentanil administered; recovery room profile, such as postoperative nausea and vomiting (PONV), and the number of patients requiring rescue analgesics; as well as postoperative hospital stay, were evaluated. Finally, postoperative laryngeal data, including POST, hoarseness, and cough, were collected.

POST, defined as pain in the larynx or pharynx at rest and upon swallowing after surgery [6], was evaluated during the patients’ PACU stay and on postoperative days (PODs) 1 and 2. The severity of POST was graded using a 0–3 scale (0 = none, no POST; 1 = mild, less severe than that with a cold; 2 = moderate, similar to that with a cold; and 3 = severe, more severe than that with a cold) [19]. The incidence of POST was defined as the number of patients with a grade of sore throat ≥ 1 over 2 PODs.

Postoperative hoarseness (PH), defined as a harsh or strained voice characterized by an altered vocal quality [20], was assessed during the PACU stay and on PODs 1 and 2 using a scale of 0–3 (0 = none; 1 = mild, noticed by the patient only; 2 = severe, evident to the observer; 3 = aphonia, loss of voice) [19]. The incidence of PH was defined as the number of patients with a grade of hoarseness ≥ 1.

The coughing of each patient was evaluated during emergence from anesthesia, including before, at, and after extubation, as well as during PACU stay. The degree of cough was divided into four grades (0 = none, 1 = single cough, 2 = more than one episode of unsustained coughing (≤5 s), and 3 = sustained coughing (>5 s)) [21].

### 2.7. Statistical Analysis

Data were analyzed using SAS version 9.4 (SAS Inc., Cary, NC, USA). The Shapiro–Wilk test was performed for the normality of continuous variables. Further, normal continuous variables are expressed as mean ± standard deviation and were analyzed using Student’s *t*-test. Other continuous variables are expressed as median (first quartile (Q1) to third quartile (Q3)) and were analyzed using the Mann–Whitney U test. Categorical variables were analyzed using the chi-square or Fisher’s exact test and are presented as the number of patients (percentage). Statistical significance was set at *p* < 0.05.

### 2.8. Ethical Statement

This retrospective study was conducted after obtaining approval from the Institutional Review Board and Hospital Research Ethics Committee of the Yonsei University Health System, Seoul, Republic of Korea (IRB protocol No. 4-2023-0154) for retrospective data collection. The requirement for informed consent was waived owing to the retrospective study design. The study was conducted in accordance with the current version of the Declaration of Helsinki.

## 3. Results

The characteristics of the 176 patients included in the final analysis are presented in Table 1. Parameters such as sex, age, BMI, ASA physical status, underlying disease, smoking history, and preoperative symptoms did not significantly differ between the two groups.

The surgical characteristics of the enrolled patients are summarized in Table 2. The number of patients who underwent bilateral, conventional, or total thyroidectomies was significantly higher in the IONM group than in the control group (*p* = 0.009, <0.001, and <0.001, respectively). The intraoperative findings indicated that tracheal invasion, thyroiditis, enlarged lymph nodes, and bleeding tendencies were higher in the IONM group than in the control group (*p* = 0.033, 0.028, 0.032, and 0.001, respectively). The proportion of cases requiring nerve sacrifice owing to RLN invasion did not significantly differ between the two groups (2% vs. 1%; *p* > 0.999). However, complications such as RLN injury did not occur in either group.

The perioperative variables are presented in Table 3. The duration of anesthesia and surgery was longer in the IONM group than in the control group (both *p* < 0.001). The number of patients with PONV or those requiring rescue analgesics in the recovery room did not significantly differ between the groups. Despite the significant difference in the length of hospitalization after surgery between the two groups, the difference was not clinically significant.

**Figure 3:**
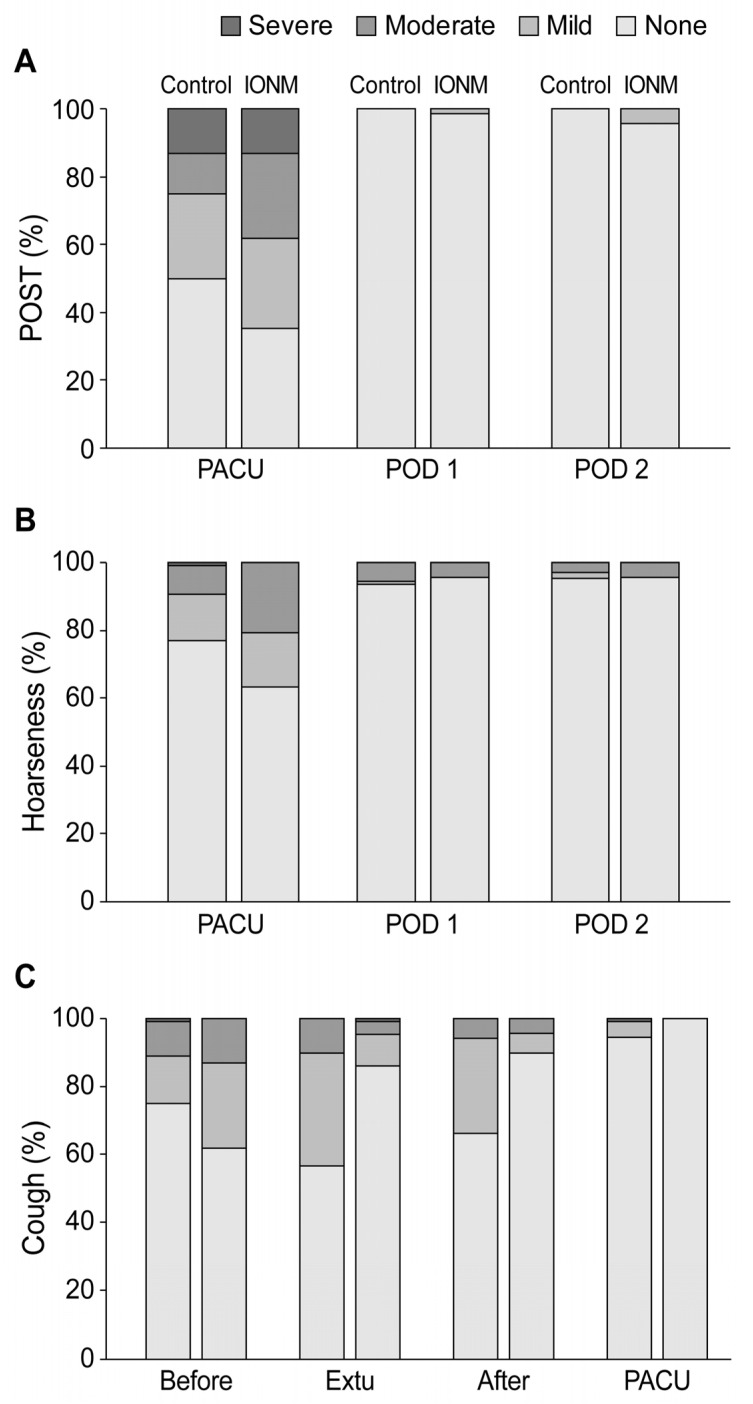
Postoperative laryngopharyngeal complications (**A**) Postoperative sore throat, (**B**) hoarseness, (**C**) cough. POST grade: none = no POST; mild = less severe than that with a cold; moderate = similar to that with a cold; and severe = more severe than that with a cold. Hoarseness grade: none = no hoarseness; mild = noticed by the patient only; moderate = evident to the observer; and severe = loss of voice. Cough grade: none = no cough; mild = single cough; moderate = more than one episode of unsustained cough; and severe = sustained and repetitive cough with head lift. IONM, intraoperative neural monitoring; POST, postoperative sore throat; PACU, post-anesthesia care unit; POD, postoperative day; Before, before extubation; Extu, at extubation; after, after extubation.

Figure 3 depicts the postoperative laryngopharyngeal complications, including POST, hoarseness, and cough. These values were not significantly different between the two groups. On POD 0 (in the PACU), POST was experienced by 65% (44/68) and 50% (54/108) of patients in the IONM and control groups, respectively. Hoarseness was observed in 37% (25/68) and 23% (25/108) of patients in the IONM and control groups, respectively, during the immediate postoperative period. Approximately 44% (47/108) and 14% (23/68) of patients in the control and IONM groups, respectively, experienced coughing at least once during the emergence process.

## 4. Discussion

Although IONM has many advantages for high-risk patients undergoing thyroidectomy, research on the effect of the ETT with laryngeal adhesive electrodes for IONM on postoperative laryngopharyngeal complications is currently limited. Despite its tendency to cause intratracheal mechanical irritation, most studies on IONM have been confined to demonstrating its usefulness. In this study, we assessed the effect of the laryngeal adhesive surface electrode that is used in IONM on laryngopharyngeal complications. To our knowledge, this is the first study to evaluate the effects of the ETT with laryngeal adhesive electrodes for IONM during thyroidectomy on postoperative laryngopharyngeal complications.

RLN palsy (RLNP) is one of the most serious complications of thyroid surgery, with the risk further increasing by up to 20% after reoperation [22,23,24]. Transient and permanent injuries to the RLN after thyroidectomy occur in 5–8% and 1–3% of cases, respectively [25,26]. RLN injury can result in various symptoms, including hoarseness, vocal changes, aspiration, and coughing or choking during swallowing. In cases of bilateral RLN injury, more severe symptoms, such as stridor, airway obstruction, and difficulty breathing, can occur [27]. IONM is widely used during thyroidectomy to prevent nerve injury. During IONM, electrodes placed on the vocal cords of the patient aid in the real-time monitoring of nerve signals to provide continuous feedback on nerve function, enabling early identification of RLN and preservation of function [1,28]. The use of IONM in thyroidectomies, especially in high-risk procedures such as reoperations and thyroidectomies for malignancy or thyrotoxicosis, is beneficial [27].

Laryngopharyngeal complications, such as POST, hoarseness, and cough, are commonly reported after thyroidectomy [20]. For example, Christensen et al. [9] and Ryu et al. [5] reported that POST was present in 62% of cases, whereas hoarseness was observed in 55–57% of cases. We observed that the incidence of POST was 65% and 50% in the control and IONM groups, respectively, during the immediate postoperative period. However, hoarseness was observed in 23% and 37% of cases in the control and IONM groups, respectively. These complications are often attributed to ETI-related factors [5,20]. In addition to trauma to the larynx or pharynx caused by ETI, manipulation of the trachea and the surrounding larynx during thyroidectomy can also play a significant role in the occurrence of these complications [8,11].

The adhesion of laryngeal surface electrodes to the ETT shaft can increase its rigidity and overall outer diameter, resulting in changes in resistance and stiffness [29]. Such differences in morphological or mechanical characteristics, when adhesive surface electrodes for IONM are used during ETI compared to using a standard ETT, might elevate the risk of mechanical irritation and exacerbate laryngeal lesions. Despite these concerns, IONM has become a widely used standard procedure during thyroidectomy and is considered inevitable. In our study, we observed no significant differences in postoperative laryngopharyngeal complications, including POST, hoarseness, and cough, between the group that received the ETT with adhesive electrodes for IONM and the control group that received a simple ETT.

In this study, data collected from our institution indicated that, compared with the control group, the IONM group had a longer surgical duration and included more complex cases, such as those with tracheal invasion, as demonstrated by the intraoperative findings. In Korea, the HIRA restricts the indications for IONM. Hence, IONM cannot be applied to all patients with thyroid cancer despite its usefulness. Consequently, the ratio of patients who underwent IONM to those who did not was different, with a higher proportion of patients undergoing MIT in the control group.

Owing to the same factor, different pathologic data were observed between the two groups. However, we noted that the IONM group, which underwent more extensive surgery with a longer duration and higher potential for tracheal stimulation than did the control group, revealed no significant differences in postoperative laryngeal complications, despite differences in surgical and pathologic data between the two groups.

In our study, RLN invasion resulted in RLN sacrifice in two patients in the control group and one in the IONM group. For the two cases in the control group, the preoperative neck sonography and intraoperative findings were discordant. Although the cancer cases were initially suspected to involve only the anterior strap muscles preoperatively, nerve involvement was confirmed during surgery, resulting in the decision to sacrifice the RLN. In contrast, one patient in the IONM group exhibited tracheoesophageal groove extension on preoperative magnetic resonance imaging. During surgery, the RLN was observed to have penetrated the thyroid cancer and adhered to the superficial esophagus. To ensure the complete removal of the cancer, the RLN was sacrificed and the esophagus was shaved. After cases of sacrificing RLN were excluded, no complications involving RLN injury were observed in the control or IONM groups.

However, this study has some limitations, such as its small sample size and retrospective nature. Furthermore, patients were classified into the control and IONM groups based on the influence of the HIRA criteria. The markedly higher number of total thyroidectomy cases in the IONM group specifically posed inherent limitations in assessing the observed differences in surgical characteristics between the two groups. Thus, it is crucial to take these limitations into consideration when interpreting the findings related to surgical outcomes, as well as when designing future studies to account for the differences in surgical procedures. Although our results indicated that the IONM group included more high-risk and complex cases and required longer surgical times, we observed no significant difference in laryngopharyngeal complications between the two groups. Further large-scale prospective trials with well-balanced groups are required to address these limitations and provide a more definitive understanding of the findings. Employing a standardized study design by conducting the same surgical procedure, such as total thyroidectomy with central node dissection, in both groups would facilitate a more direct comparison between the groups and improve the validity of the study results. Nevertheless, this study could serve as a foundation for future prospective studies and has clinical significance in mitigating concerns regarding the potential impact of monitoring electrodes on laryngopharyngeal complications.

## 5. Conclusions

The ETT with laryngeal adhesive electrodes for IONM during thyroidectomy did not affect the incidence and severity of postoperative laryngopharyngeal complications, including POST, hoarseness, and cough. Our findings suggest that the electrodes used for IONM during thyroidectomy can be safely utilized without increasing the risk of laryngopharyngeal complications. Further prospective, double-blinded, randomized clinical trials are required to gain a clearer understanding.

## Figures and Tables

**Table 1:** Patient characteristics.

	Control (*n* = 108)	IONM (*n* = 68)	*p*-Value
Sex (F:M, F(%))	78:30 (72%)	51:17 (75%)	0.685
Age, years	48 ± 13	49 ± 14	0.846
BMI, kg/m^2^	24.4 ± 4.5	24.9 ± 4.3	0.396
ASA I/II/III	24/69/15	9/47/12	0.308
Underlying disease			
Chronic cough	1 (1%)	1 (1%)	>0.999
HTN	23 (21%)	15 (22%)	0.905
DM	8 (7%)	8 (12%)	0.328
Smoking history			0.578
Nonsmoker	94 (87%)	56 (82%)	
Previous smoker	11 (10%)	8 (12%)	
Current smoker	3 (3%)	4 (6%)	
Preoperative symptom			
Incidentaloma	108 (100%)	66 (97%)	0.148
Compression	0 (0%)	0 (0%)	>0.999
Hoarseness	0 (0%)	0 (0%)	>0.999

Data are presented as the mean ± standard deviation or number of patients (proportion). BMI, body mass index; HTN, hypertension; DM, diabetes mellitus; ASA, American Society of Anesthesiologists; IONM, intraoperative neural monitoring.

**Table 2:** Surgical characteristics.

	Control (*n* = 108)	IONM (*n* = 68)	*p*-Value
Cancer location			0.009 *
Right	54 (50%)	23 (34%)	
Left	38 (35%)	22 (32%)	
Bilateral	16 (15%)	23 (34%)	
Type of operation			<0.001 *
Conventional thyroidectomy	66 (61%)	66 (97%)	
Minimally invasive thyroidectomy	42 (39%)	2 (3%)	
Type of thyroidectomy			<0.001 *
Lobectomy	73 (68%)	24 (35%)	
Ipsilateral total and contralateral partial	18 (17%)	2 (3%)	
Total thyroidectomy	17 (16%)	42 (63%)	
Co-operation with parathyroidectomy	1 (1%)	4(6%)	0.070
Intraoperative findings			
Recurrent laryngeal nerve invasion/sacrifice	2 (2%)	1 (1%)	>0.999
Strap muscle invasion	3 (3%)	2 (3%)	>0.999
Tracheal invasion	1 (1%)	5 (7%)	0.033 *
Esophageal invasion	0 (0%)	1 (1%)	0.386
Thyroiditis	39 (36%)	36 (53%)	0.028 *
Enlarged lymph node	25 (23%)	26 (38%)	0.032 *
RLN injury	0 (0%)	0 (0%)	>0.999

Data are presented as the number of patients (proportion). * *p* < 0.05, IONM, intraoperative neural monitoring; RLN, recurrent laryngeal nerve.

**Table 3:** Perioperative characteristics.

	Control (*n* = 108)	IONM (*n* = 68)	*p*-Value
Duration of anesthesia, min	94 ± 19	110 ± 25	<0.001 *
Duration of operation, min	71 ± 20	89 ± 25	<0.001 *
Endotracheal tube size, cm	6.5 [6.5–7.5]	6.5 [6.5–8.0]	0.132
Total fluid intake, mL	528 ± 135	538 ± 163	0.646
Blood loss, mL	1 ± 3	7 ± 14	0.001 *
Intraoperative administered remifentanil, μg	0.4 [0.3–0.4]	0.4 [0.35–0.5]	<0.001 *
Recovery room profile			
PONV	2 (2%)	4 (6%)	0.207
Rescue analgesics, *n*	9 (8%)	5 (7%)	>0.999
Postoperative hospital stays, days	3 [2–3]	3 [3–3]	<0.001 *

Data are presented as the mean ± standard deviation, median [q1–q3], or number of patients (proportion). * *p* < 0.05, IONM, intraoperative neural monitoring; PACU, post-anesthesia care unit; PONV, postoperative nausea and vomiting.

## Data Availability

The original contributions presented in the study are included in the article. Further inquiries can be directed to the corresponding authors.
